# Resected case of giant cystic thymoma with spontaneous intracystic hemorrhage

**DOI:** 10.1186/s40792-019-0586-9

**Published:** 2019-02-19

**Authors:** Yasuto Sakaguchi, Teruya Komatsu, Yasutaka Takubo, Yasuji Terada

**Affiliations:** 1 Department of Thoracic Surgery, Kishiwada City Hospital, 1001 Gakuhara-cho, Kishiawada-shi, Kishiwada city, Osaka 596-8501 Japan; 2 Department of Thoracic Surgery, Nagara Medical Center, 1300-7 Nagara, Gifu city, Gifu Japan; 3 Department of Thoracic Surgery, Nagahama City Hospital, 313 Ooinui-cho, Nagahama city, Shiga Japan; 4 Department of Thoracic Surgery, Respiratory Disease Center, Kyoto Katsura Hospital, 17 Yamadahirao-cho Nishikyouku, Kyoto, Japan

**Keywords:** Hemorrhage, Cysts, Mediastinal tumor, Cystic thymoma

## Abstract

**Background:**

Spontaneous intracystic hemorrhage of cystic thymoma is very rare. We encountered a patient with giant cystic thymoma with spontaneous intracystic hemorrhage and successfully resected the thymoma.

**Case presentation:**

A 38-year-old man was referred to our hospital with chest pain. Computed tomography revealed a uniform anterior mediastinum cystic mass. Two days after hospitalization, his chest pain worsened. Subsequent computed tomography showed that the tumor had become inhomogeneous. The patient’s symptoms gradually improved over a fortnight, and surgery was performed. The tumor was a cystic mass with a thick fibrous capsule filled with hemorrhagic necrotic tissue and was diagnosed as a cystic thymoma.

**Conclusions:**

Mediastinal cystic lesion with expansion or contrasting effects in the wall may be a cystic thymoma, and it has the possibility of hemorrhaging in the cyst. In such a case, surgical resection is recommended.

## Background

Few studies have reported cases of cystic thymoma with complete cystic degeneration, and these cases rarely involve intracystic hemorrhage [1]. To the best of our knowledge, no reports have visually documented the occurrence of hemorrhage within a cystic thymoma. Here, we report a case of a giant cystic thymoma that spontaneously developed intracystic hemorrhage after patient admission. During hospitalization, diagnostic imaging showed that the mass had changed in size and the cyst wall had thickened. The mass was successfully resected and diagnosed as cystic thymoma with intracystic hemorrhage.

## Case presentation

**Fig. 1:**
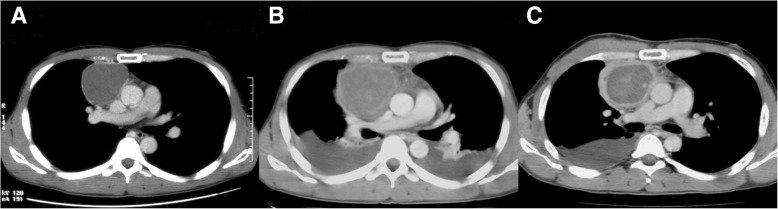
**a** Enhanced chest computed tomography scan before hospitalization showing the giant cystic mediastinal tumor. The inside of the cyst is homogeneous, and the surroundings are enhanced. **b** Enhanced chest computed tomography scan on hospitalization day 2 showing the expansion and heterogenization of the cystic tumor with bilateral pleural effusion. **c** Enhanced chest computed tomography scan on hospitalization day 14 showing the reduction of pleural effusions, thickening of the cystic capsule, reduction of the tumor’s size, and re-homogenization of the cystic tumor

A 38-year-old man visited our hospital complaining of anterior chest pain. He had no significant medical or family history, and the vital signs were stable. Ischemic events were not observed in electrocardiography, but chest X-ray and computed tomography (CT) showed a cystic lesion (6.0 × 7.0 × 10.0 cm) in the anterior mediastinum (Fig. 1a). Although the cystic capsule demonstrated contrast enhancement, its fluid component had low radiation absorbance. Based on these findings, we suspected the mass to be a thymic cyst. Blood tests indicated the presence of inflammation (white blood cell count 11,200/μL and C-reactive protein 3.38 mg/dL).

Two days after hospitalization, the patient developed dyspnea and his chest pain worsened. Subsequent chest CT showed that the cystic lesion had become inhomogeneous and the radiation absorbance of the cyst’s fluid component had increased (Fig. 1b). The cyst wall became thickened, and bilateral effusion was observed. Blood tests indicated that hemoglobin levels had decreased from 15.8 to 12.8 g/dL, and levels of inflammatory markers had increased, with the fever exceeding 38.5 °C. Needle aspiration biopsy and tumor wall biopsy with a small skin incision were performed; however, we could not obtain a diagnosis. One week after admission, general condition and laboratory data of the patient gradually improved. A chest CT on day 13 showed that the tumor had become small in size with a thickened wall (Fig. 1c). The effusion on the right side had decreased and that on the left side had disappeared.

**Fig. 2:**
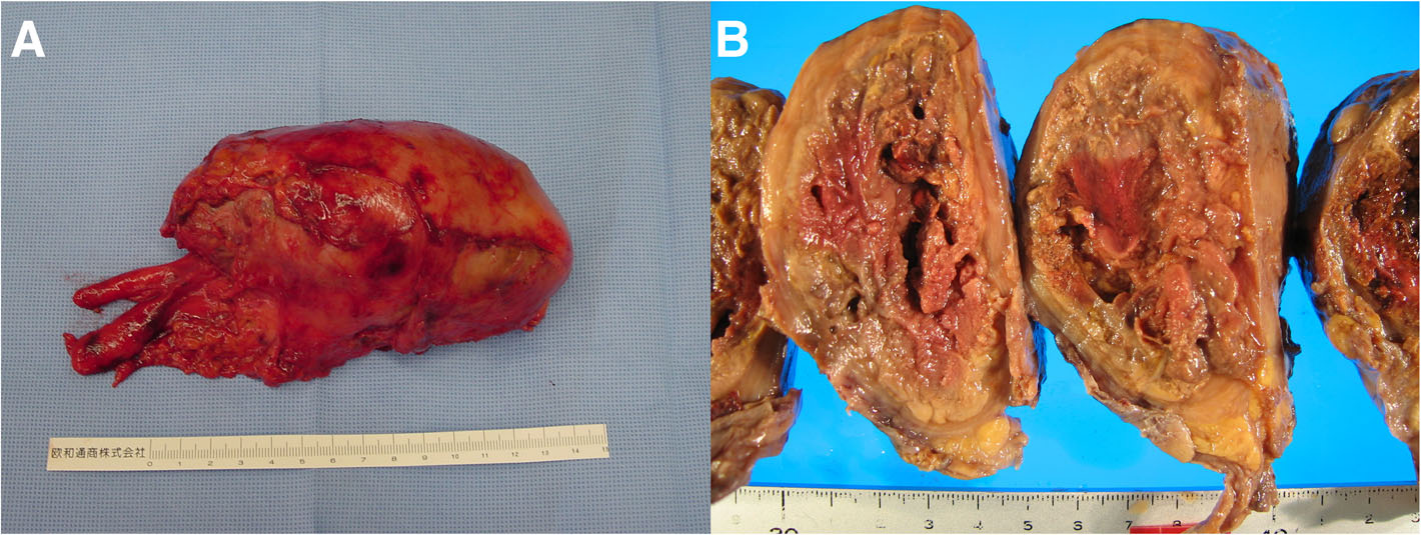
**a** Macroscopic appearance of the resected tumor. **b** Macroscopic appearance of the tumor’s cut surface

The patient had recovered enough to undergo surgery; the tumor was resected by sternotomy on day 18. The tumor was found to be encased in a smooth, yellow, and elastic coat. The tumor was densely adhered to the junction of the left brachiocephalic vein and superior vena cava, and it was required to detach the tumor from the dense adhesion site carefully. The right phrenic nerve was preserved, and the right pleural effusion was serous. The tumor and thymic tissue were resected en bloc. The operative time was 288 min, and the estimated blood loss was 521 mL. The resected tumor was covered with a thick, fibrous capsule, and the lumen was filled with necrotic tissue and hemorrhagic material (Fig. 2a, b). The postoperative course was uneventful, and he was discharged on day 26.

**Fig. 3:**
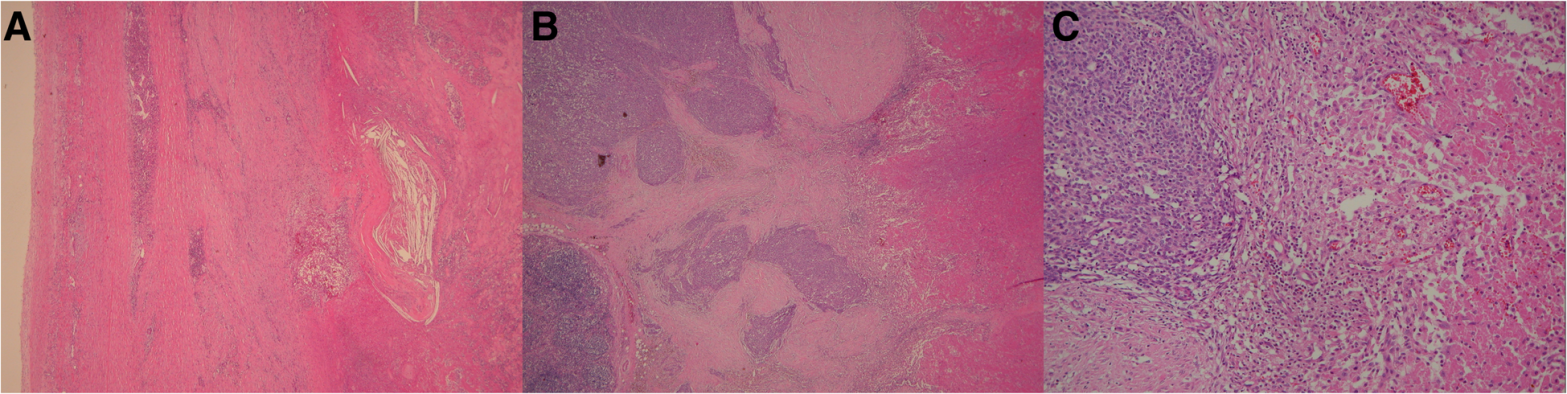
**a**, **b** The pathological findings showed a fibrotic cyst wall; the cyst was filled with necrotic tissue. The slight proliferation of lymphocytes was confirmed in the necrotic tissue and around the cyst wall (hematoxylin and eosin staining). **c** The tumor was diagnosed as type B1 cystic thymoma

The pathological findings showed a fibrotic cyst wall; the cyst was filled with necrotic tissue. The slight proliferation of lymphocytes was confirmed in the necrotic tissue and around the cyst wall (Fig. 3a, b). The tumor was diagnosed as type B1 cystic thymoma (Fig. 3c). As the tumor did not appear to have spread beyond the capsule, it was determined to be at Masaoka stage I. Nevertheless, the dense adherence of the tumor to its surrounding tissue indicated the possibility of invasion, and postoperative radiotherapy (50 Gy) was administered.

Two years after the surgery, recurrent metastasis of the tumor was found on the right pleura and the left upper lobe of the lung. The patient was treated with chemotherapy, radiotherapy, and local resection. The patient remains alive 12 years after the first surgery. Following an analysis of the tissue obtained from the resected recurrent tumor, the pathological diagnosis was changed to type B3 thymoma.

## Discussion

We described a case of a giant cystic thymoma showing rapid morphological changes with clinical symptoms. After the patient’s general condition stabilized, the tumor was excised and diagnosed as cystic thymoma.

Although the cystic degeneration of thymomas is relatively frequent (approximately 40%), thymoma of an entire cystic lesion like the one in our case is rare [1]. Dyer was the first to describe a case of cystic thymoma in which the lumen became complete cystic degeneration [2]. In our patient, the thymoma had originally presented as a thin-walled cyst. After inflammatory changes, however, the cyst wall thickened but the thymic tissue remained. Despite the sparse cellular component, the tissue that remained in the cyst was found to be thymic tissue. The mass was therefore diagnosed as a cystic thymoma.

Moran and Suster have postulated that chronic inflammation in thymomas affects vascular blood supply, which results in infarction, necrosis, and hemorrhage [3]. This can eventually cause cystic changes in the thymoma and may explain the presence of hematomas, hemorrhagic material, and necrotic tissue within these tumors. In contrast, some cystic thymomas are reported to have serous cystic components [2, 4]. These cases may occur when a thymoma develops and proliferates on the wall of the original thymic cyst. Studies have reported cases where a thymoma was confirmed to be part of the cyst wall [5, 6].

An interesting feature of this case is that although the cyst’s fluid component demonstrated low radiation absorbance in the initial CT scan, the absorbance increased after the patient’s chest pain worsened. The cyst had increased in size and its wall had thickened. This was followed by a reduction in the cyst’s size, further thickening of the cyst wall, and an improvement in symptoms. These changes were likely triggered by the serous cystic thymoma causing a hemorrhagic infarction by obstructing blood flow. Hemorrhage at the infarct site would have increased the radiation absorbance of the thymoma’s fluid component. The decrease in hemoglobin was the result of intracystic bleeding. Inflammation after the reabsorption of cystic hemorrhage and the shrinking of the cyst may explain the cyst wall thickening. Because the right pleural effusion collected during surgery was serous, the cause of bilateral pleural effusion that occurred in the preoperative course was not the tumor perforation, but rather increased permeability of the mediastinal pleura with mediastinal inflammation. Considering the causes of a series of major symptoms, the chest pain during admission may have been caused by an increasing size of the cystic thymoma and the severe chest pain may have been caused by the hemorrhagic infarction.

From the viewpoint of treatment, it is preferable to perform surgery when the patient exhibits a better general condition, with less inflammation, in order to obtain the biopsy result. However, an emergent operation remains useful, if exacerbation of symptoms occurs. Notably, the hospitalization period might be shortened by performing an emergency operation.

## Conclusion

Mediastinal cystic lesion with expansion or contrasting effects in the wall may be a cystic thymoma, and it has the possibility of hemorrhaging in the cyst. In such a case, surgical resection is recommended.

## Abbreviation

CT: Computed tomography
